# *Mycobacterium tuberculosis* Beijing genotype family strain isolated from naturally infected plateau zokor (*Myospalax baileyi*) in China

**DOI:** 10.1038/emi.2017.33

**Published:** 2017-06-07

**Authors:** Lin Sun, Xiang Chen, Wenhai Zhang, Gencheng Huang, Yuanyuan Zhang, Zhengzhong Xu, Baofa Yin, Wanhong Wei, Xinan Jiao, Kanglin Wan

**Affiliations:** 1 Jiangsu Key Laboratory of Zoonosis/Jiangsu Co-Innovation Center for Prevention and Control of Important Animal Infectious Diseases and Zoonoses, Yangzhou University, Yangzhou 225009, China; 2 State Key Laboratory for Infectious Disease Prevention and Control, National Institute for Communicable Disease Control and Prevention, Chinese Center for Disease Control and Prevention, Beijing 102206, China; 3 Department of Bioscience, College of Bioscience and Biotechnology, Yangzhou University, Yangzhou 225009, China

**Dear Editor,**

*Mycobacterium tuberculosis* (MTB) remains one of the world’s most successful pathogens, infecting one-third of humans. In 2015, there were an estimated 10.4 million new MTB cases in humans, leading to 1.4 million deaths.^1^ Wild animals can act as reservoirs and sources of MTB infections for humans and livestock, and this public health hazard can affect global economies. Understanding the host species involved in zoonotic pathogen maintenance and transmission is essential to prevent the diseases caused by such pathogens.

Tuberculosis caused by MTB is common among dogs^2, 3^ and among elephants^4^ and may also be transmitted to humans. A similar situation possibly exists for wild rodents that carry *M. microti*^5^ and other *Mycobacterium* species,^6^ but MTB has not been found in them so far.

Although rodents are rarely clinically affected by mycobacteriosis, they can serve as potential reservoirs of MTB for wildlife, domestic animals and humans.^6^ Here we aimed to investigate the prevalence of mycobacteria in the wild plateau zokor (*Myospalax baileyi*), a rodent species endemic in China. The plateau zokor is a blind subterranean mole rat that spends its life underground in sealed burrows.^7^ This highly specialized subterranean herbivore is broadly distributed across farms, prairies, alpine prairies and meadows.^8^

In May 2010, a total of nine wild plateau zokors with no clinical signs of mycobacteriosis were caught in the Haibei Alpine Meadow Ecosystem Research Station, Northwest Institute of Plateau Biology, Chinese Academy of Sciences, Qinghai Province, China (37°29′–37°45′ N, 101°12′–101°33′ E, 3200 m above sea level). All procedures involved in the handling and care of the animals were approved by the China Zoological Society.

The animals were immediately sealed in separate sterile sample bags after being humanely killed in Qinghai, that is to say, each animal was placed in a sample bag, and then transported on ice to the Chinese CDC Tuberculosis Reference Laboratory. All the experiments were completed in an ABL-2 laboratory. After dissection, the lungs were removed, homogenized and decontaminated, and then the treated homogenate samples were inoculated onto modified Lowenstein–Jensen solid selective medium and meanwhile cultured in MGIT 960 liquid medium. All the samples screened in this study were smeared and stained using the Ziehl–Neelsen method.^9^

Of the nine samples, only samples 1, 8 and 9 had positive test results. Samples 1 and 8 were shown to be positive by Ziehl–Neelsen staining, Lowenstein–Jensen culturing and MGIT 960 culturing. For sample 9, the MGIT 960 culture was positive, while the other two tests were negative. We then inoculated the MGIT 960 products from sample 9 onto Lowenstein–Jensen slants for further bacterial isolation. Finally, three isolates, namely, JSA11006, JSA11010 and JSA11028, were successfully cultured. Using *p*-nitrobenzoic acid (PNB)/thiophene-2-carboxylic acid hydrazide (TCH)-differentiated media, JSA11006 and JSA11028 were categorized as nontuberculous mycobacterial, while JSA11010 was categorized as MTB (Table 1).

The *Mycobacterium* colonies cultured on Lowenstein–Jensen slants were scraped and re-suspended in 100 μL of TE buffer (pH 8.0), and then heat inactivated at 80 °C for 30 min. Genomic DNA was then extracted from them using the conventional cetyltrimethyl ammonium bromide (CTAB) method.^10^ Multi-locus polymerase chain reaction involving seven target genes (*16S rRNA*, *Rv0577*, *IS1561′*, *Rv1510*, *Rv1970*, *Rv3877/8* and *Rv3120*)^11^ confirmed classification of the three isolates as nontuberculous mycobacterial and MTB (Table 1). Using *16S rRNA* and *rpoB* gene sequencing (Table 1), JSA11006 was identified as *M. setense*, and JSA11028 was identified as *M. septicum*. Spoligotyping of the MTB strain was performed as previously described.^12^ Using the SITVITWEB database^13^ (http://www.pasteur-guadeloupe.fr:8081/SITVIT_ONLINE/index.jsp), JSA11010 was identified as belonging to the Beijing family with Spoligo International Type number=1, and its variable number of tandem repeats profile was 2-3-4-3-4-3-3-4-3-7-4-4-4-2-5-1-5-3-3-4-4-6-3-3, as defined by the number of tandem repeats in MIRU02, Mtub04, ETRC, MIRU04, MIRU40, MIRU10, MIRU16, Mtub21, MIRU20, QUB11b, ETRA, Mtub29, Mtub30, ETRB, MIRU23, MIRU24, MIRU26, MIRU27, Mtub34, MIRU31, Mtub39, QUB26, QUB4156 and MIRU39.

Drug susceptibility testing of the MTB isolate was performed using the Lowenstein–Jensen proportion method recommended by the World Health Organization.^14^ The critical concentrations were 0.2 μg/mL for isoniazid, 40 μg/mL for rifampicin, 4 μg/mL for streptomycin, 2 μg/mL for ethambutol, 4 μg/mL for ofloxacin, 40 μg/mL for capreomycin and 40 μg/mL for prothionamide. H37Rv was used as a reference strain. JSA11010 was found to be sensitive to all the tested drugs.

In conclusion, *Mycobacterium* isolates were obtained from three of nine plateau zokors, and one strain, which was identified as MTB, belongs to the Beijing genotype family. To the best of our knowledge, this is the first report of an MTB infection in plateau zokor, and this species may be an important reservoir for MTB and a zoonotic threat to humans and other animals. The underground channels used by plateau zokors are shared by other wild animals such as steppe polecats (*Mustela eversmanni*) and plateau pikas (*Ochotona curzoniae*). These animals spend their lives both underground and overground, so they have the opportunity to come into contact with humans, livestock and wild animals infected with MTB. In addition, plateau zokors usually live underground, but they have short-term overground activities during pregnancy when they may come into contact with infected animals or people. Therefore, it is possible that MTB could be transmitted to plateau zokors in this way.

Although rodents are resistant to mycobacteriosis, they do have the potential to carry or transmit the mycobacteria present in the environment to humans and other animals. Targeted surveillance is necessary to monitor the presence and spread of MTB, as well as the efficacy of the control measures in operation.

## Figures and Tables

**Table 1:** Identification of *Mycobacterium* species by PNB/TCH testing, multi-locus PCR and sequencing of *16S rRNA* and *rpoB* genes

**Strain number**	**PNB**	**TCH**	**L–J**	**Result**	**Multi-locus PCR**	**Sequencing-*****16S rRNA***	**Identity**	**Sequencing-*****rpoB***	**Identity**
JSA11006	+	+	+	NTM	NTM	*M. setense*	1210/1214 (99%)	*M. setense*	720/720 (100%)
JSA11010	−	+	+	MTB	MTB				
JSA11028	+	+	+	NTM	NTM	*M. septicum*	1363/1366 (99%)	*M. septicum*	732/735 (99%)

Abbreviations: *p*-nitrobenzoic acid, PNB; thiophene-2-carboxylic acid hydrazide, TCH; polymerase chain reaction, PCR; Lowenstein–Jensen, L–J; nontuberculous mycobacteria, NTM; *Mycobacterium tuberculosis*, MTB.

+ represents growth, − represents no growth.
